# Correction: The impact of Fosetyl-Aluminium application timing on Karnal bunt suppression and economic returns of bread wheat (*Triticum aestivum* L.)

**DOI:** 10.1371/journal.pone.0259550

**Published:** 2021-10-28

**Authors:** Muhammad Arif, Sagheer Atta, Muhammad Amjad Bashir, Muhammad Ifnan Khan, Ansar Hussain, Muhammad Shahjahan, Mona S. Alwahibi, Mohamed Soliman Elshikh

The following information is missing from the Funding statement: Authors would like to extend their sincere appreciation to the Researchers Supporting Project number (RSP-2020/173), King Saud University, Riyadh, Saudi Arabia.
